# Rapid Geographical Discrimination of Wenzhou Ougan via GC-IMS: A Comparison of Peel and Juice Volatile Fingerprints

**DOI:** 10.3390/s26175351

**Published:** 2026-08-24

**Authors:** Yiping Tang, Zibo Chen, Ziyi Huo, Tianyu Shi, Xuezhen Hong

**Affiliations:** College of Quality and Standardization, China Jiliang University, Hangzhou 310018, China; p24190855010@cjlu.edu.cn (Y.T.); p24190855013@cjlu.edu.cn (Z.C.); 17816736022@163.com (Z.H.); 2401502105@cjlu.edu.cn (T.S.)

**Keywords:** GC-IMS, ougan mandarin, volatile organic compounds, geographical origin traceability, variable importance in projection (VIP)

## Abstract

As a National Geographical Indication (GI) product, Wenzhou Ougan (WZ) is difficult to distinguish visually from mandarins of other origins. In this study, 34 peel samples and 47 juice samples from WZ and three external origins were analyzed by headspace gas chromatography–ion mobility spectrometry (HS-GC-IMS), and their VOC fingerprints were compared. A total of 59 and 68 VOC signal variables were extracted from peel and juice samples, respectively; compound assignments were tentative annotations based on retention index and drift time matching with the GC-IMS library. Gallery plots and two-dimensional spectra revealed distinct differences in several VOC signal regions among origins. Principal component analysis (PCA), partial least squares discriminant analysis (PLS-DA), and variable importance in projection (VIP) values were used to evaluate grouping trends and screen discriminant VOC signal variables. For WZ-origin samples, the main WZ-high discriminant VOC signal variables in peel included furfuryl alcohol, 3-octanone, pentanal, n-nonanal, ethyl 2-methylbutyrate, butyl hexanoate, alpha-pinene, and 3-methylbutyric acid, while those in juice mainly included beta-ocimene, limonene, furfuryl alcohol, 2-acetylfuran, 2-methylfuran, butyric acid, and pentanoic acid. These findings indicate that integrated VOC fingerprints show potential for rapid geographical discrimination of Wenzhou Ougan, while further independent validation and complementary chemical confirmation are required.

## 1. Introduction

Ougan mandarin (*Citrus reticulata* cv. *Suavissima*) is a representative local citrus cultivar in Wenzhou, Zhejiang Province, China, and has been recognized as a geographical indication product. The national standard GB/T 22442-2008 specifies its protected production area, quality requirements and testing methods, providing a basis for standardized production and quality evaluation of Wenzhou Ougan [1]. Compared with common citrus cultivars, Ougan has distinctive regional cultivar characteristics and high utilization value. Previous studies have shown that Ougan and its peel are rich in flavonoids and polymethoxyflavones and may exhibit biological activities such as antiproliferative activity, regulation of glucose/lipid metabolism, and modulation of gut microbiota [2,3,4]. These studies indicate the nutritional and functional value of Ougan and provide a scientific basis for its further development and utilization.

The protection and authentication of geographical indication agricultural products have received increasing attention in recent years. Consumers and industries have placed higher demands on the authenticity and traceability of products with clear geographical origins, making origin discrimination an important issue in food quality control and brand protection [5,6]. Wenzhou Ougan is difficult to distinguish visually from mandarins produced in other regions. This may affect the brand value of Wenzhou Ougan as a geographical indication product and may also weaken consumer confidence. Therefore, establishing a rapid, objective, and visually interpretable analytical approach for identifying Wenzhou-origin Ougan has practical significance for protecting local specialty agricultural products and supporting market supervision.

Volatile organic compounds (VOCs) are closely related to citrus cultivar, tissue type, maturity, growing environment and postharvest handling, and they can reflect metabolic and flavor-related differences among samples. Previous studies have demonstrated the potential of VOCs or metabolomic profiles in citrus classification and origin discrimination. For example, volatile compounds and a mass spectrometry-based electronic nose have been used to distinguish the geographical origin of oranges [7], while metabolomic approaches have been used to compare chemical profiles among citrus varieties [8]. Integrated HS-GC-IMS and UPLC-Q-Orbitrap HRMS have also been applied to reveal volatile and nonvolatile metabolite differences among different citrus peels [9]. In addition, volatile aroma components in citrus peels have been used to distinguish different cultivation lines or citrus species [10,11]. These studies provide important references for citrus VOC analysis and origin-related discrimination.

Gas and VOC detection technologies differ in their sensing principles, information outputs, and applicable analytical tasks. An electronic nose usually integrates an array of partially selective sensors with pattern-recognition algorithms and is suitable for rapid and relatively low-cost classification of overall odor patterns. However, it does not chromatographically separate individual VOCs, and its performance may be influenced by sensor cross-sensitivity, sensor drift, humidity, and sensor-array configuration [12]. Conductometric metal-oxide semiconductor sensors measure gas-induced changes in the electrical resistance or conductance of the sensing material. Such sensors can achieve high sensitivity and selectivity for specific analytes, as demonstrated for acetic anhydride and ammonia, while their sensing performance is closely related to surface oxygen chemistry and gas–surface interactions [13,14,15]. Fiber-optic gas sensors convert gas-induced changes in optical properties into analytical signals and offer advantages such as resistance to electromagnetic interference and suitability for remote sensing. However, they are also commonly developed for one or a limited number of target gases, such as hydrogen [16]. Therefore, sensor-based techniques are particularly suitable for target-gas monitoring or rapid overall odor-pattern screening, but they generally do not provide chromatographic resolution of numerous VOC signals in a complex food headspace.

Chromatographic techniques provide a different level of analytical information because volatile components are separated before detection. GC-MS combines gas-chromatographic separation with mass-spectral detection and provides stronger structural identification and quantitative capabilities, making it more suitable for high-confidence compound confirmation and accurate quantification. HS-GC-IMS combines gas-chromatographic retention separation with ion-mobility separation and generates two-dimensional VOC fingerprints based on retention time and drift time. Compared with a conventional electronic-nose response pattern, HS-GC-IMS provides more resolved information on multiple VOC signal variables, while offering relatively simple headspace preparation, high sensitivity, rapid analysis, and intuitive fingerprint visualization [17]. However, GC-IMS library matching provides tentative rather than definitive compound identification, and important discriminant signals should be further confirmed using GC-MS and authentic standards. Thus, these techniques should be regarded as complementary: electronic noses and target-specific sensors are suitable for rapid screening or selected-gas monitoring, HS-GC-IMS is suitable for rapid non-targeted fingerprint comparison of complex samples, and GC-MS is more suitable for definitive chemical confirmation and quantification.

Citrus peel and juice may provide different VOC information. Peel is rich in essential-oil-related volatiles and often contains abundant terpenes, aldehydes and esters, which are important for citrus aroma and sample differentiation [9,10,11]. In contrast, juice VOC profiles may be influenced by tissue matrix, pulp composition, maturity and processing-related factors [18]. Therefore, peel and juice may show different response patterns in HS-GC-IMS analysis. Simultaneously comparing peel and juice can help clarify how different tissue matrices contribute to the identification of Wenzhou-origin Ougan. This comparison is important because practical origin discrimination requires not only an effective classification model but also interpretable VOC fingerprint information.

HS-GC-IMS is an established analytical technique for food VOC fingerprinting [17]. Previous citrus-related HS-GC-IMS studies have mainly focused on VOC profiling for purposes other than geographical origin discrimination. For example, HS-GC-IMS has been used to characterize disease-related VOC changes in navel orange and pomelo leaves [19] and processing-related volatile changes in finger citron [20], while integrated HS-GC-IMS and UPLC-Q-Orbitrap HRMS have been applied to compare metabolite profiles among different citrus peels [9]. In contrast, previous applications of HS-GC-IMS to geographical discrimination have mainly involved other regional or geographical-indication foods, such as huajiao and winter jujube [21,22]. Thus, although HS-GC-IMS has been established for citrus VOC profiling and regional food discrimination, to the best of our knowledge, no previous study has specifically applied HS-GC-IMS to distinguish Wenzhou-origin Ougan from external origins or systematically compared peel and juice as alternative matrices for this task. Therefore, the contribution of the present study lies in addressing the specific geographical-discrimination problem of Wenzhou Ougan and in systematically evaluating the respective VOC fingerprint information provided by peel and juice, rather than in the HS-GC-IMS technique itself.

In this study, mandarin samples from Wenzhou and three external origins, including Guangxi, Fujian and Sichuan, were analyzed by HS-GC-IMS. Peel and squeezed juice samples were prepared and analyzed separately. GC-IMS fingerprints, relative response values, Welch’s ANOVA, principal component analysis (PCA), partial least squares discriminant analysis (PLS-DA), permutation tests and variable importance in projection (VIP) values were used to evaluate origin-related VOC differences. The objectives of this study were to (1) characterize VOC fingerprint differences in peel and juice samples from different origins; (2) screen discriminant VOC signal variables contributing to the differentiation between Wenzhou-origin Ougan and non-Wenzhou samples; and (3) compare the applicability of peel and juice samples for supporting Wenzhou-origin discrimination.

## 2. Materials and Methods

### 2.1. Sample Collection and Preparation

**Figure 1:**
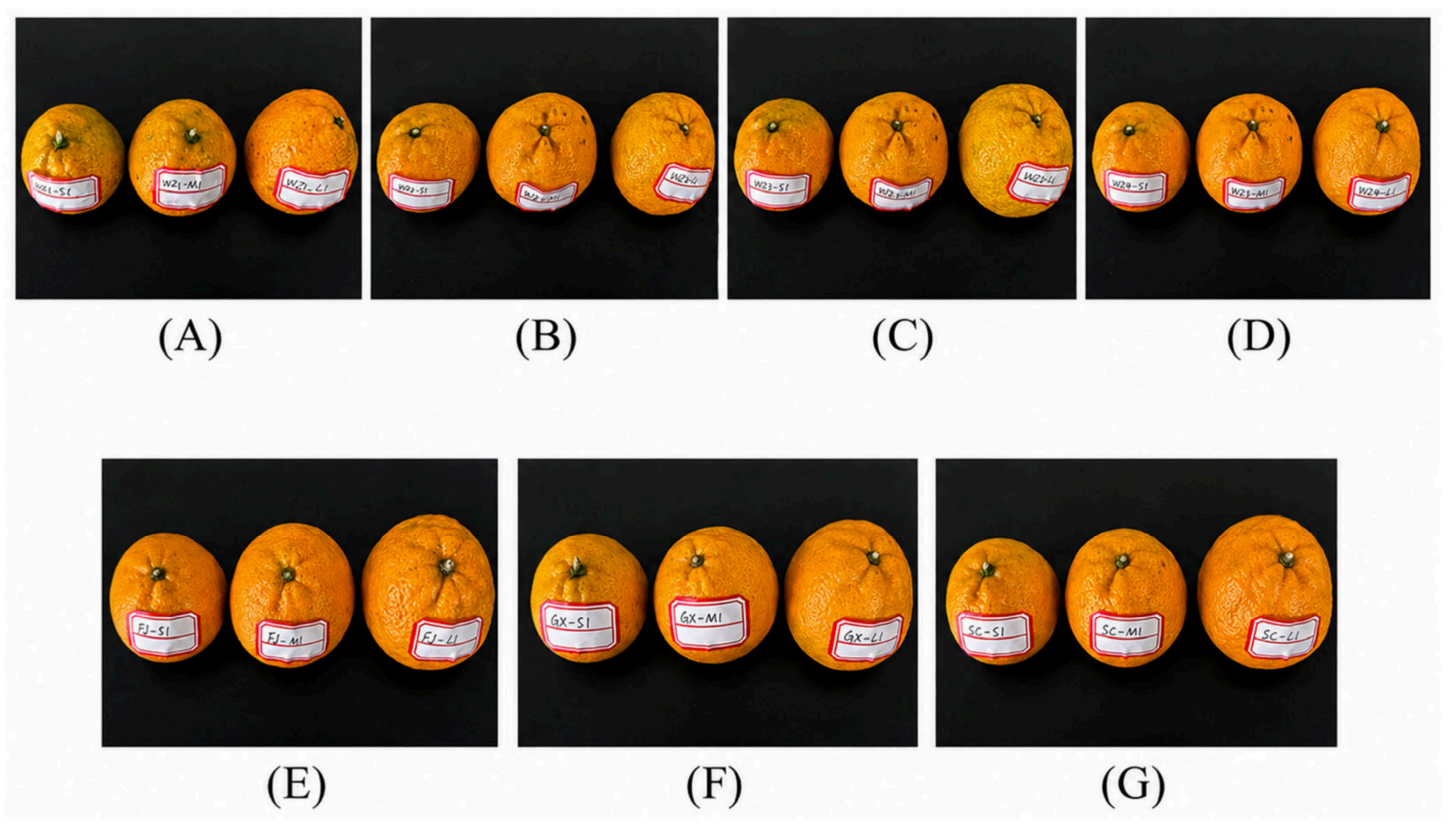
Representative photographs of Ougan samples from different sampling locations and geographical origins. (**A**) Zeya, Ouhai, Wenzhou (WZ1); (**B**) Sanyang Street, Ouhai, Wenzhou (WZ2); (**C**) Xianyan Liqing Farm, Ouhai, Wenzhou (WZ3); (**D**) Xianyan Bingqing Farm, Ouhai, Wenzhou (WZ4); (**E**) Fujian (FJ); (**F**) Guangxi (GX); (**G**) Sichuan (SC). Within each panel, S, M, and L denote representative small, medium, and large fruits, respectively.

Mandarin samples were collected from WZ and three external origins, including GX, FJ and SC. To improve the representativeness of Wenzhou-origin samples, WZ samples were collected from different orchards within the Wenzhou Ougan protection area. The WZ samples were coded as WZ1 (Zeya, Ouhai), WZ2 (Sanyang Street, Ouhai), WZ3 (Xianyan Liqing Farm, Ouhai) and WZ4 (Xianyan Bingqing Farm, Ouhai), while the external-origin samples were coded as GX, FJ and SC. Fruits with similar maturity and without visible disease, insect damage or mechanical injury were selected. The harvest dates were kept as similar as possible. Representative photographs of Ougan samples from the different sampling locations and geographical origins are shown in Figure 1. Small, medium, and large fruits are included to illustrate the within-origin variation in external fruit size.

Peel and juice samples were prepared separately. For juice preparation, the pulp was squeezed to obtain the juice, which was then homogenized and used directly for HS-GC-IMS analysis without further filtration or centrifugation. For peel preparation, the outer peel was manually removed from the fruit, cut into small pieces, and homogenized before HS-GC-IMS analysis. Because peel and juice represent different tissue matrices, they were analyzed independently by HS-GC-IMS, and separate response matrices were constructed for subsequent statistical analysis. In total, 47 juice samples and 34 peel samples were obtained. The sample sources and codes are shown in Table 1.

### 2.2. HS-GC-IMS Conditions

The analysis was performed using a FlavorSpec^®^ gas chromatography-ion mobility spectrometer (G.A.S., Dortmund, Germany) equipped with a CTC-PAL 3 static headspace autosampler. An MXT-5 capillary column (15 m × 0.53 mm, 1.0 μm) was used for chromatographic separation. For each analysis, 1.0 g of homogenized peel or 10 mL of homogenized juice was transferred into a 20 mL headspace vial for HS-GC-IMS analysis. The headspace injection volume was 500 μL. The incubation time was 15 min, the incubation temperature was 50 °C, the injection needle temperature was 85 °C, the incubation rotation speed was 500 r/min, and splitless injection was used.

The column temperature was maintained at 60 °C, and the total running time was 30 min. High-purity nitrogen (purity ≥ 99.999%) was used as the carrier gas. The carrier gas flow was programmed as follows: the initial flow rate was 2 mL/min and maintained for 0–2 min, increased to 10 mL/min from 2 to 10 min, increased to 100 mL/min from 10 to 20 min, and maintained at 100 mL/min from 20 to 30 min.

The IMS conditions were as follows: high-purity nitrogen (purity ≥99.999%) was used as the drift gas; a tritium source (^3^H) was used as the ionization source; the drift tube length was 53 mm; the electric field strength was 500 V/cm; the drift tube temperature was 45 °C; the drift gas flow rate was 150 mL/min; and the instrument was operated in positive ion mode.

### 2.3. Data Processing and Statistical Analysis

Raw GC-IMS data were processed using the supporting software packages of the instrument, including Laboratory Analytical Viewer (LAV, version 2.2.1) and Library Search (version 1.0.3). The Reporter and Gallery Plot functions used for data visualization and analysis are integrated modules within LAV. GC-IMS fingerprints, two-dimensional spectra and peak response matrices were generated separately for peel and juice samples. VOC signal variables were tentatively annotated by matching retention index (RI) and drift time (DT) information with the built-in NIST and IMS databases.

All subsequent statistical analyses, chemometric modeling, and data visualization were performed in Python (version 3.12.7), using NumPy (version 1.26.4), pandas (version 2.2.2), SciPy (version 1.13.1), scikit-learn (version 1.5.1), and Matplotlib (version 3.9.2).

Relative response values were obtained from normalized GC-IMS peak volumes and expressed as mean ± standard deviation. Differences in VOC relative response values among geographical origins were evaluated using Welch’s one-way ANOVA followed by the Games–Howell post hoc test, considering the unequal sample sizes among groups [23,24]. A value of *p* < 0.05 was considered statistically significant. According to their dominant structural features and functional groups, the VOC signal variables were classified into chemical classes, including esters, alcohols, aldehydes, ketones, acids, terpenes and terpenoids, furans and furan derivatives, aromatic compounds and others.

The normalized VOC response matrices were subjected to PCA and PLS-DA. PCA was used as an unsupervised method to visualize the overall distribution and grouping trends of samples from different origins. PLS-DA was used as a supervised method to evaluate the origin discrimination ability of peel and juice VOC fingerprints. The PLS-DA models were evaluated using R^2^Y and Q^2^ values, and model robustness was further assessed using 200 label-permutation tests [25,26]. In the permutation tests, class labels were randomly permuted while the response matrix remained unchanged, and the original model statistics were compared with those obtained from the permuted models.

To further evaluate whether the geographical discrimination performance depended on the choice of classifier, PLS-DA was additionally compared with support vector machine (SVM) and random forest (RF) classifiers using the same VOC response matrices. All three classifiers were evaluated using repeated stratified five-fold cross-validation with 10 repeats, and identical training and test partitions were used for each algorithm. Classification accuracy was used as the common performance metric. The SVM used a radial basis function kernel with C = 1.0 and gamma = “scale”, while the RF model consisted of 500 decision trees with the square root of the number of features considered at each split. Data standardization for PLS-DA and SVM was performed within each training fold to avoid information leakage. No additional feature selection or hyperparameter optimization was performed during the algorithm comparison.

VIP values were calculated from the PLS-DA models to evaluate the contribution of each VOC signal variable to origin discrimination [27]. Variables satisfying both VIP > 1 and *p* < 0.05 were selected as discriminant VOC signal variables for WZ-origin discrimination. These variables were further interpreted according to their group mean responses and Games–Howell post hoc results. Because GC-IMS annotation was based on RI and DT database matching, the screened variables were interpreted as VOC signal variables rather than fully confirmed single-compound markers.

## 3. Results and Discussion

### 3.1. GC–IMS Gallery Plot Fingerprint Analysis

GC-IMS Gallery Plot fingerprints were constructed separately for peel and juice samples to visually compare the VOC profiles of Ougan samples from different geographical origins. In the Gallery Plot, each column represents a VOC signal variable, each row represents a sample, and the color intensity reflects the relative response intensity of the corresponding signal peak. Therefore, the Gallery Plot provides an intuitive overview of VOC fingerprint differences among samples.

**Figure 2:**
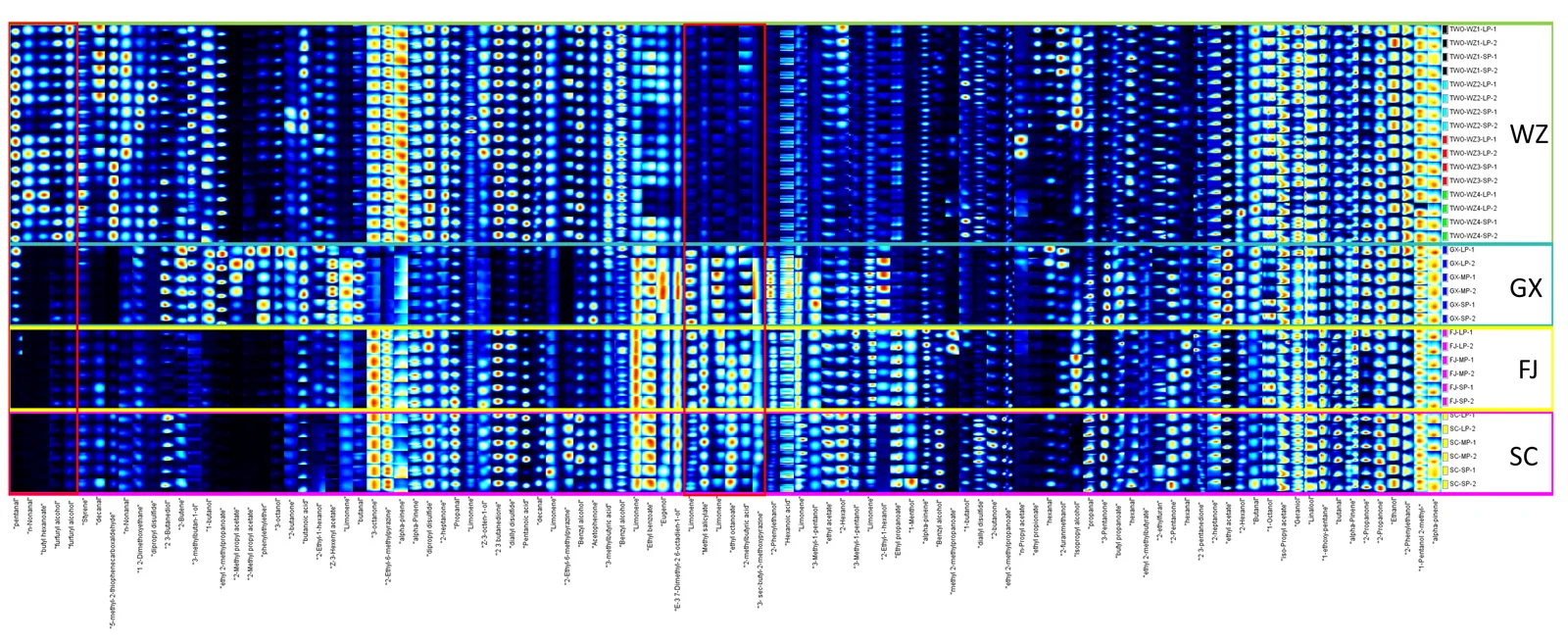
GC-IMS fingerprint for Ougan peel samples collected from different origins.

As shown in Figure 2, peel samples from different origins showed visible differences in their GC-IMS fingerprints. The red-boxed regions correspond to representative VOC signal regions showing clear response differences among origins. In peel samples, these differential regions were relatively concentrated and were mainly reflected between WZ samples and samples from external origins. Several representative signal regions tentatively assigned to pentanal, n-nonanal-related variables, furfuryl alcohol-related variables, butyl hexanoate, limonene-related variables, methyl salicylate and ethyl octanoate showed clear response differences among origins. These results suggest that peel fingerprints provide intuitive visual evidence for distinguishing Wenzhou-origin Ougan from non-WZ samples.

**Figure 3:**
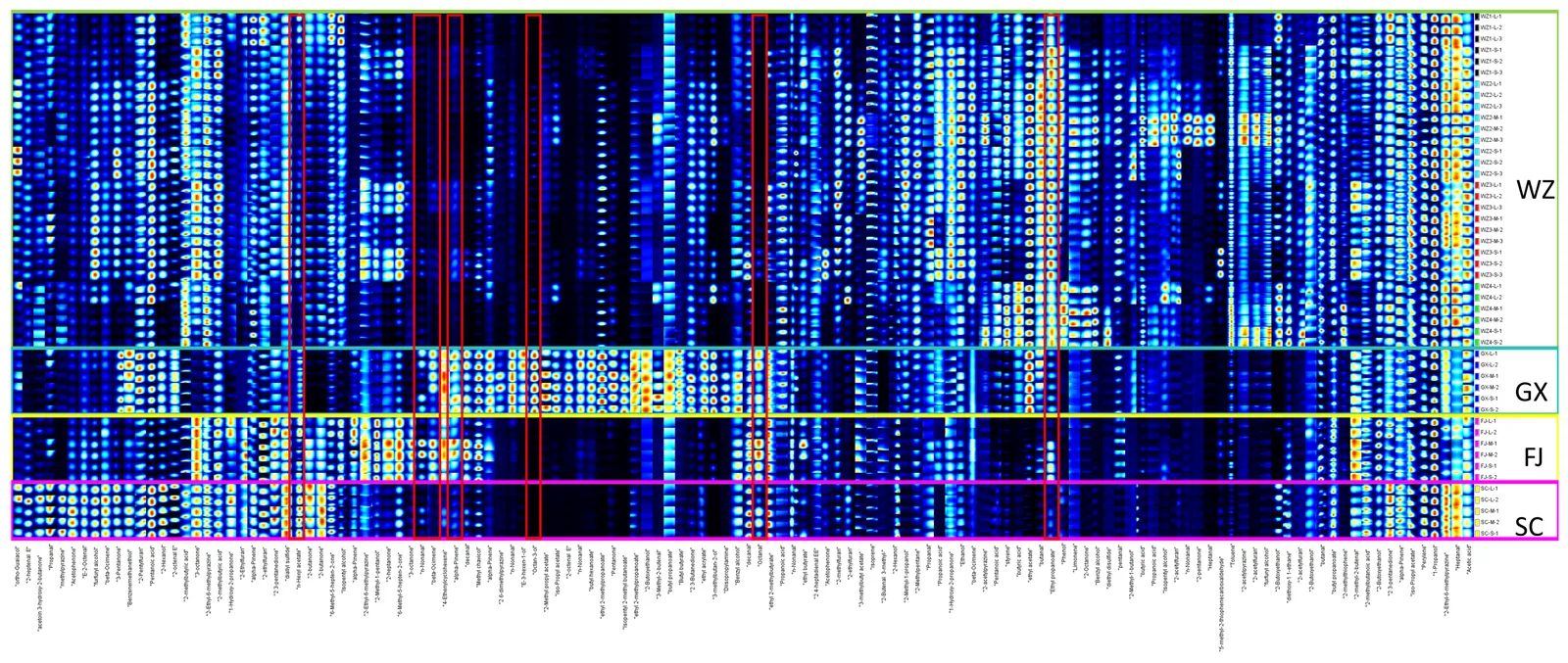
GC-IMS fingerprint for Ougan juice samples collected from different origins.

As shown in Figure 3, juice samples also exhibited origin-related differences in the Gallery Plot fingerprints. Compared with peel samples, the highlighted differential regions in juice samples were more dispersed across the fingerprint. Representative signal regions tentatively assigned to ethyl acetate, octan-3-ol, n-nonanal-related variables, alpha-pinene, beta-ocimene, octanal and n-hexyl acetate showed visible response differences among origins. These results indicate that juice fingerprints can provide complementary information for differentiating WZ and non-WZ samples, although the response pattern was more distributed than that observed in peel samples.

**Figure 4:**
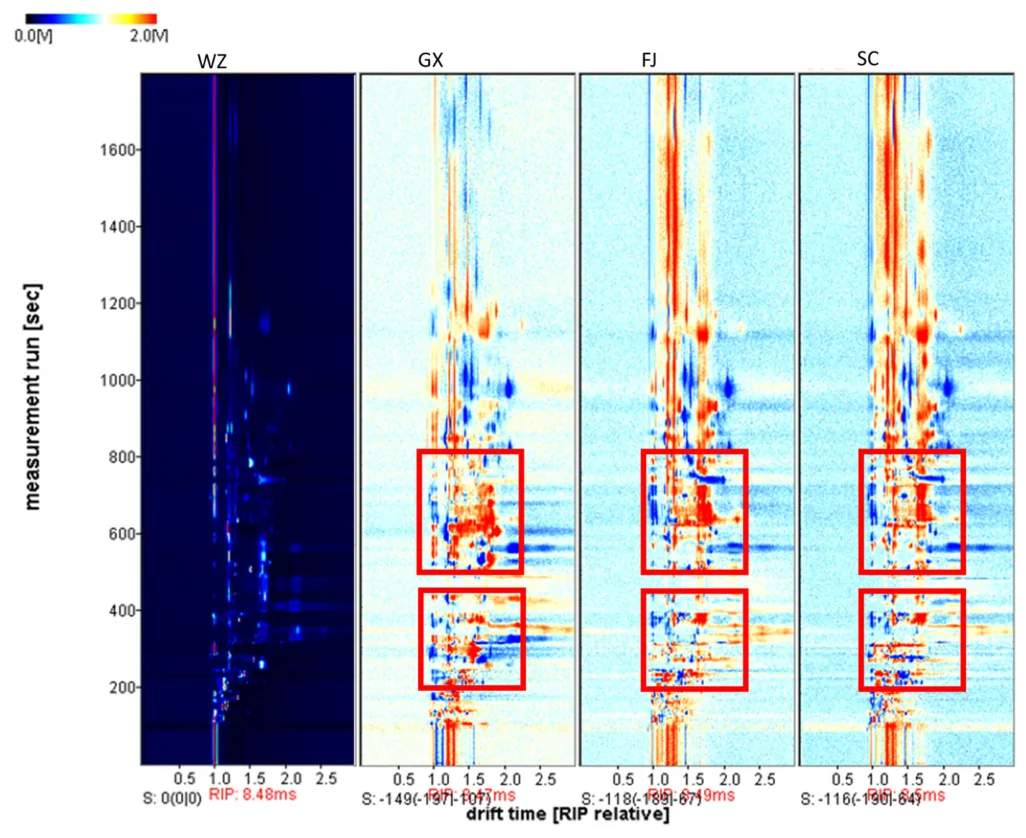
Two-dimensional GC-IMS spectra of representative Ougan peel samples from different origins.

**Figure 5:**
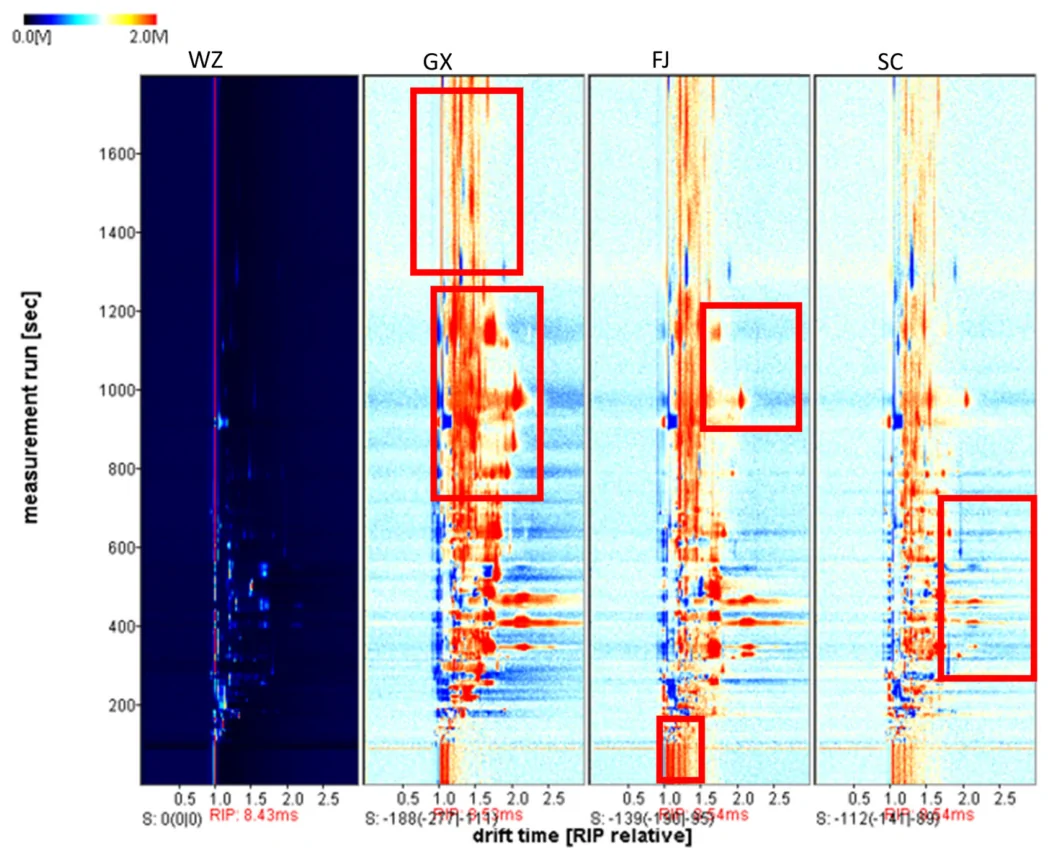
Two-dimensional GC-IMS spectra of representative Ougan juice samples from different origins.

Figure 4 and Figure 5 show the two-dimensional GC-IMS spectra of representative peel and juice samples from different origins, respectively. In these spectra, the red-boxed regions indicate representative differential signal regions in the retention time and drift time dimensions. For peel samples, the highlighted regions were mainly concentrated in several relatively limited spectral areas, indicating that peel VOC differences were relatively localized. In contrast, the highlighted regions in juice samples were distributed across a broader spectral range, indicating a more dispersed multivariable response pattern. These results provide preliminary qualitative evidence for origin-related VOC fingerprint differences, which were further evaluated by relative response analysis and multivariate statistical analysis.

### 3.2. Tentative Annotation of VOC Signal Variables and Quantitative Analysis of Relative Responses

**Figure 6:**
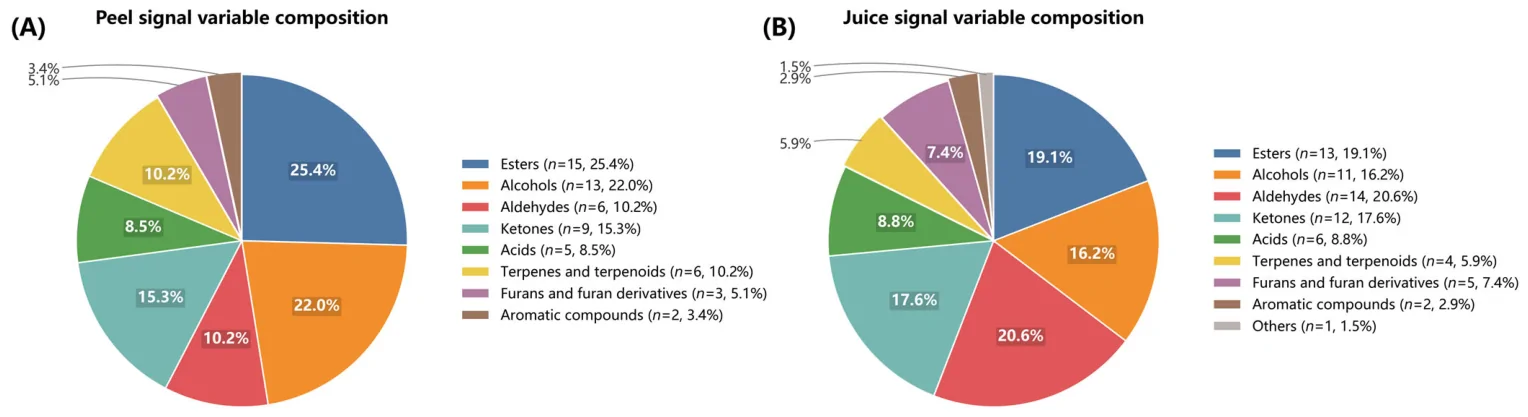
Chemical-class composition of VOC signal variables in Ougan samples: (**A**) peel samples; (**B**) juice samples.

Before comparing individual VOC signal variables, the chemical-class composition of the VOC signal variables listed in Table 2 and Table 3 was summarized (Figure 6). A total of 59 VOC signal variables were listed for peel samples and 68 VOC signal variables were listed for juice samples. These variables were classified into nine chemical classes according to their dominant structural features and functional groups, including esters, alcohols, aldehydes, ketones, acids, terpenes and terpenoids, furans and furan derivatives, aromatic compounds, and others.

In peel samples, esters accounted for the largest proportion of VOC signal variables, followed by alcohols, ketones, aldehydes, terpenes and terpenoids, acids, furans and furan derivatives, and aromatic compounds. No “others” variable was listed in peel samples. In juice samples, aldehydes represented the largest class, followed by esters, ketones, alcohols, acids, furans and furan derivatives, terpenes and terpenoids, aromatic compounds, and others. These results indicate that peel and juice samples differed not only in their GC-IMS response patterns but also in the distribution of VOC signal classes. Peel samples contained relatively more ester- and alcohol-related variables, whereas juice samples showed a higher proportion of aldehyde- and ketone-related variables.

To further characterize the volatile signals corresponding to the differential GC-IMS regions, VOC signal variables in peel and juice samples were tentatively annotated using the GC-IMS library, and their relative response values were calculated. Table 2 and Table 3 summarize the RI, RT, DT and relative response values of VOC signal variables in peel and juice samples from WZ, GX, FJ and SC. The data are expressed as mean ± standard deviation. Different lowercase letters within the same row indicate significant differences among geographical origins based on Welch’s one-way ANOVA followed by the Games–Howell post hoc test. The response values represent normalized GC-IMS peak-volume intensities rather than absolute concentrations. Because the compound assignments were based on retention index and drift time matching with the GC-IMS library, the listed variables should be regarded as tentatively annotated VOC signal variables rather than definitively identified compounds or confirmed chemical markers. Complementary GC-MS analysis, preferably together with authentic standards, would provide higher confidence in the chemical identities of the most important discriminant signals.

In peel samples (Table 2), the origin-related differences were mainly reflected in aldehyde-related, furan-related, terpene-related, and aromatic-related signals rather than in uniform changes across all VOC classes. WZ peel showed relatively stronger responses for several aldehyde-related and furan-related variables, including pentanal, n-nonanal, decanal, furfuryl alcohol, and 2-ethylfuran, whereas several terpene-related, ester-related, and alcohol-related variables showed higher responses in external-origin samples. Fruit VOCs are derived from multiple biochemical pathways, including fatty acid, amino acid, and terpenoid metabolism, and these pathways jointly contribute to the formation of aldehydes, alcohols, esters, ketones, and terpenoids [28]. Citrus peel is particularly rich in essential-oil-related volatiles, especially terpenoids, while non-terpenoid aldehydes, alcohols, esters, and acids also contribute substantially to the peel volatile profile [29]. Therefore, the differences observed in WZ peel are more appropriately interpreted as coordinated changes among multiple volatile-related pathways rather than as enrichment of a single diagnostic compound.

In juice samples (Table 3), origin-related variation was distributed across a broader range of VOC classes than in peel. WZ juice showed relatively stronger responses for several terpene-related, furan-related, acid-related, and aromatic-related variables, including beta-ocimene, limonene, furfuryl alcohol, 2-acetylfuran, and pentanoic acid, whereas several ester-related, aldehyde-related, alcohol-related, and ketone-related variables showed higher responses in external-origin samples. Similar to other fruit matrices, juice VOCs may originate from several metabolic pathways involving fatty acids, amino acids, and terpenoid precursors [28]. Consequently, the geographical differences observed in juice are unlikely to be represented by a single chemical class; rather, they reflect a broader multivariable volatile pattern formed by signals with different biochemical origins.

Overall, the geographical differences in Ougan VOC fingerprints were associated with coordinated variation across multiple VOC classes rather than with a single compound or chemical class. Peel showed a relatively concentrated WZ-related response pattern, whereas juice exhibited broader multivariable variation. These findings indicate that geographical discrimination is supported by the overall configuration of multiple VOC signals with different biochemical origins rather than by any single VOC signal variable.

The origin-related VOC differences observed in this study may reflect the combined influence of environmental conditions, cultivation practices, fruit physiological status, and tissue-specific metabolism. Environmental factors associated with different production regions, such as temperature, light exposure, and water availability, can influence fruit metabolism and consequently affect the formation and accumulation of volatile compounds. Environmental conditions and postharvest factors have been reported to influence fruit volatile composition [28]. Cultivation-related factors may further affect fruit physiological status and volatile composition; for example, rootstock selection has been reported to significantly influence the volatile profile of mandarin juice [30]. In addition, fruit maturity and postharvest conditions can alter volatile composition [18,28]. Peel and juice also differ substantially in tissue structure and metabolic characteristics. Citrus peel is rich in oil-gland-associated volatile compounds, particularly terpenoids, whereas juice sacs exhibit a distinct volatile composition [29,31]. Therefore, the differences observed among WZ, GX, FJ, and SC samples should be interpreted as origin-associated fingerprint differences arising from multiple interacting environmental and biological factors rather than as effects caused by geographical location alone. Because these factors were not independently controlled in the present study, their individual contributions cannot be determined from the current dataset and should be further investigated using multi-year and multi-site sampling designs.

### 3.3. Principal Component Analysis of VOC Fingerprints

To further evaluate the overall distribution characteristics of VOC fingerprints from different geographical origins, PCA was performed using the VOC signal variables listed in Table 2 and Table 3. PCA is an unsupervised dimensionality reduction method that projects high-dimensional data into a lower-dimensional space while retaining the main variance information. Therefore, it can provide an intuitive visualization of similarities and differences among samples from different origins.

**Figure 7:**
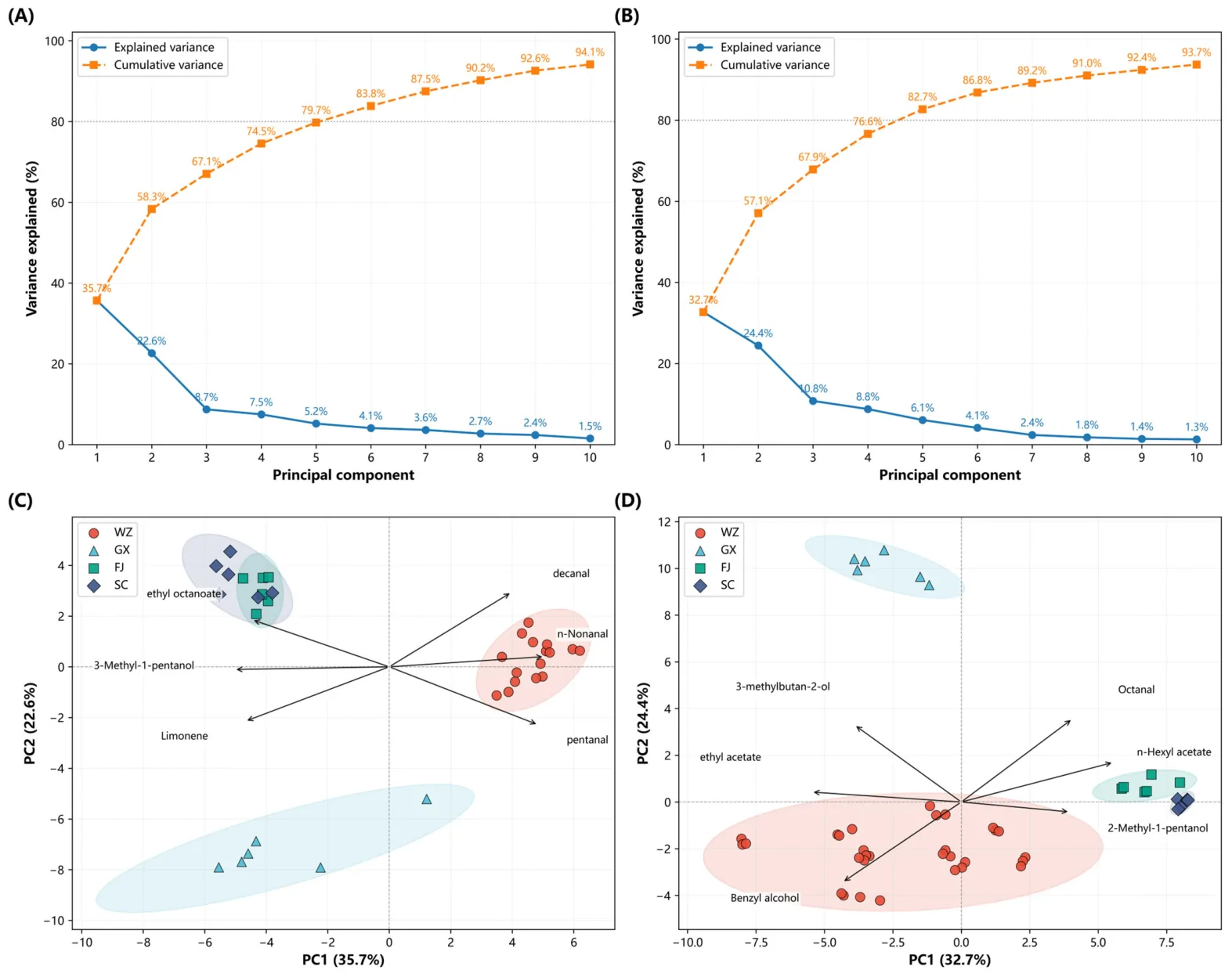
PCA of GC–IMS fingerprints of Ougan peel and juice samples. (**A**) Explained and cumulative variance plots of PCA for peel samples. (**B**) Explained and cumulative variance plots of PCA for juice samples. (**C**) PCA biplot of peel samples. (**D**) PCA biplot of juice samples. The labeled variables are representative VOC signal variables with relatively high contributions to the separation of different origins.

As shown in Figure 7A,B, the first two principal components of peel samples explained 58.33% of the total variance, with PC1 and PC2 accounting for 35.68% and 22.64%, respectively. For juice samples, PC1 and PC2 explained 32.67% and 24.42% of the total variance, respectively, with a cumulative contribution of 57.09%. These results indicate that the first two principal components retained the major variation in the VOC fingerprints of both peel and juice samples.

In the PCA score plot of peel samples (Figure 7C), WZ samples were mainly distributed on one side of PC1, while samples from external origins showed different distribution patterns along PC1 and PC2. This suggests that peel VOC fingerprints contained clear origin-related information for distinguishing WZ samples from non-WZ samples. The biplot further showed that several representative VOC signal variables, such as pentanal, benzyl alcohol, hexanal and limonene, contributed strongly to the PC1 direction. These variables were closely associated with the separation trend of peel samples from different origins.

In the PCA score plot of juice samples (Figure 7D), the four origin groups also showed visible separation tendencies, but the distribution pattern was more dispersed than that of peel samples. WZ samples were mainly distributed toward one side of PC1, whereas some non-WZ samples tended to be located in different regions of the PC1–PC2 space. Representative variables such as limonene, beta-ocimene, ethyl acetate, n-hexyl acetate, alpha-pinene and 2-methyl-2-butenal contributed to the separation along PC1. These results indicate that juice-based origin differences were reflected by broader multivariable response patterns.

Overall, PCA showed that both peel and juice VOC fingerprints contained origin-related variation. Peel samples exhibited a relatively concentrated separation pattern, suggesting that peel fingerprints provided more intuitive information for distinguishing WZ samples from non-WZ samples. Juice samples showed more dispersed but still distinguishable distribution characteristics, indicating that juice fingerprints could provide complementary information for origin discrimination. Since PCA is an unsupervised method, these results mainly provide visual evidence of sample grouping trends and should be further supported by supervised classification analysis.

### 3.4. Supervised Classification and Screening of Discriminant VOC Signal Variables

To further evaluate the origin discrimination ability of VOC fingerprints, PLS-DA was performed based on the VOC signal variables listed in Table 2 and Table 3. Unlike PCA, PLS-DA is a supervised classification method that uses class information to maximize the separation among predefined groups. Since Wenzhou Ougan is a geographical indication product, the interpretation of the PLS-DA results focused not only on the classification of the four origins but also on the differentiation of WZ samples from non-WZ samples.

**Figure 8:**
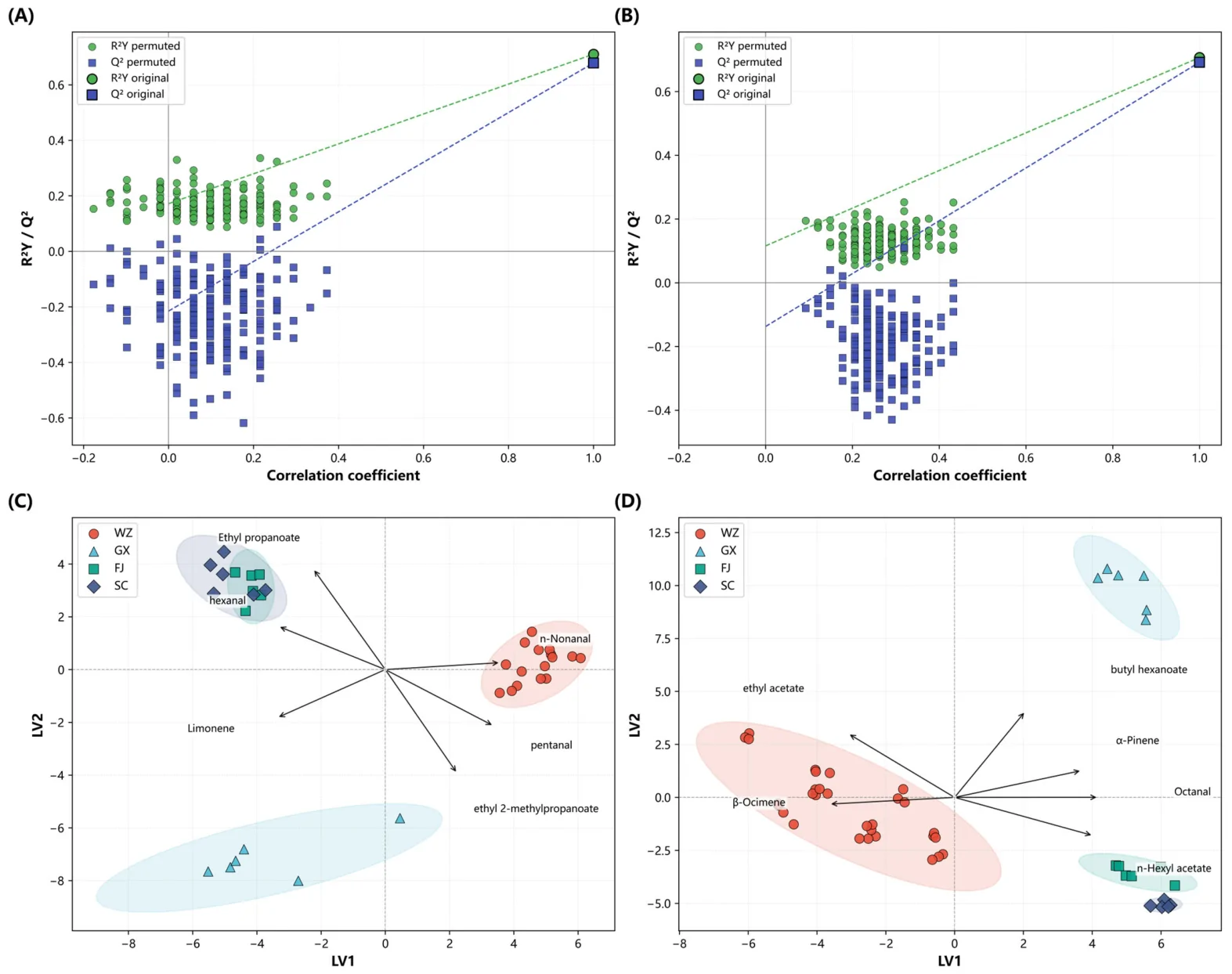
Statistical validation and biplot visualization of PLS-DA models based on GC–IMS fingerprints. (**A**) Permutation test of the peel PLS-DA model based on 200 label permutations. (**B**) Permutation test of the juice PLS-DA model based on 200 label permutations. (**C**) PLS-DA biplot of peel samples. (**D**) PLS-DA biplot of juice samples. The labeled variables are representative VOC signal variables with relatively high contributions to origin discrimination in the LV1–LV2 space.

As shown in Figure 8 and Table 4, both peel and juice PLS-DA models were established using two latent variables. The peel model achieved an R^2^Y of 0.710 and a Q^2^ of 0.678, while the juice model achieved an R^2^Y of 0.707 and a Q^2^ of 0.692. These results indicate that both models had acceptable explanatory and predictive ability for origin discrimination. Compared with the peel model, the juice model showed a slightly higher Q^2^ value, suggesting that juice VOC fingerprints provided useful predictive information in the current PLS-DA model. The permutation tests further showed that the R^2^Y and Q^2^ values of the original models were higher than those obtained from the permuted models, indicating that the observed discrimination was not mainly caused by random class assignment.

The PLS-DA score plots showed clearer group separation than the PCA score plots. In peel samples, WZ samples were separated from most non-WZ samples along the latent variable space, suggesting that peel VOC fingerprints contained useful information for identifying Wenzhou-origin Ougan. In juice samples, WZ, GX, FJ and SC samples also showed distinct distribution tendencies, indicating that juice VOC fingerprints also contributed to origin discrimination. Therefore, both peel and juice samples provided discriminative information, but their response patterns and model contributions were different.

To further examine whether the observed geographical discrimination depended on the choice of classifier, PLS-DA was compared with SVM and RF using repeated stratified five-fold cross-validation (Table 5). For peel samples, the classification accuracy increased from 0.790 ± 0.068 for PLS-DA to 0.983 ± 0.069 for SVM and 0.997 ± 0.020 for RF. For juice samples, PLS-DA achieved an accuracy of 0.873 ± 0.037, whereas both SVM and RF reached 1.000 ± 0.000 under the present internal cross-validation procedure. These results indicate that the geographical information contained in the VOC fingerprints can be effectively captured by different supervised classifiers, with SVM and RF showing stronger classification performance than PLS-DA in the present dataset. Nevertheless, given the limited sample size and the absence of an independent external validation set, the very high internal cross-validation accuracy of SVM and RF should be interpreted cautiously and requires confirmation using larger independent datasets. PLS-DA was retained as the primary chemometric model for subsequent interpretation because its latent-variable representation, loadings, and VIP values facilitate examination of the contribution of individual VOC signal variables to geographical discrimination.

The present results are consistent with previous studies showing that VOC fingerprints combined with multivariate analysis can provide useful information for geographical-origin discrimination. Centonze et al. [7] discriminated oranges from three geographical origins using an HS-SPME/MS-based electronic nose and compared PCA/LDA, SELECT/LDA, and PLS-DA models, with SELECT/LDA showing the highest predictive performance. Feng et al. [21] combined E-nose, HS-GC-IMS, and HS-SPME-GC-MS to characterize eight geographical-indication huajiao samples. Their GC-MS- and GC-IMS-based PLS-DA models effectively differentiated samples from different origins, and VIP analysis was further used to screen potential discriminant volatiles. Similarly, Qiao et al. [22] combined GC-IMS, HS-SPME-GC/MS, and E-nose with multivariate analysis to characterize volatile differences among winter jujubes from eight regions. Compared with these studies, the present work focuses specifically on Wenzhou Ougan, systematically compares peel and juice as alternative sample matrices, and further evaluates PLS-DA, SVM, and RF under the same repeated cross-validation framework. Because the sample matrices, geographical categories, analytical platforms, sample sizes, and validation strategies differ among studies, direct numerical comparison of classification accuracy across studies is not appropriate. Nevertheless, these studies collectively support the usefulness of multivariable VOC fingerprints combined with chemometric or machine-learning methods for geographical-origin discrimination.

**Figure 9:**
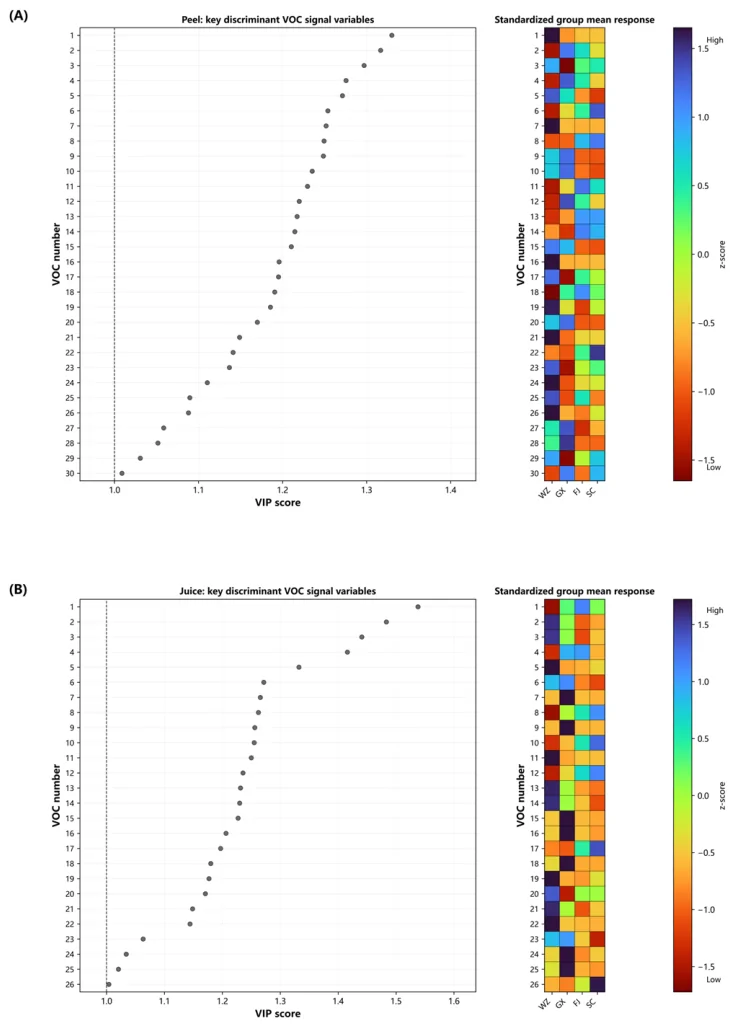
VIP scores and standardized response heatmaps of screened discriminant VOC signal variables selected by VIP > 1 and *p* < 0.05. (**A**) Peel samples; (**B**) juice samples. The left panels show VIP values calculated from the PLS-DA models, and the right panels show standardized group mean responses across WZ, GX, FJ and SC. Heatmap values are row-wise z-scores of group mean responses. The numbers correspond to the screened discriminant VOC signal variables listed in Table 6.

VIP values were calculated to evaluate the contribution of each VOC signal variable to the PLS-DA model. Variables with VIP > 1 and *p* < 0.05 were selected as discriminant VOC signal variables for WZ-origin discrimination. As summarized in Table 4, 33 peel variables and 29 juice variables had VIP values greater than 1, while 30 peel variables and 26 juice variables simultaneously satisfied VIP > 1 and *p* < 0.05. These variables are listed in Table 6 and visualized in Figure 9. In Figure 9, the VIP scores indicate the contribution of each selected variable to the PLS-DA model, whereas the heatmap shows the standardized group mean responses among WZ, GX, FJ and SC. Therefore, Figure 9 and Table 6 together reveal not only which variables contributed to the model, but also which origin showed relatively higher responses for each variable.

For peel samples, 30 VOC signal variables satisfied the combined criterion of VIP > 1 and *p* < 0.05 (Table 6 and Figure 9). These variables were distributed across multiple chemical classes, indicating that WZ-origin discrimination was supported by an integrated VOC fingerprint rather than by one dominant compound class. Representative WZ-associated variables included furfuryl alcohol, 3-octanone, pentanal, n-nonanal, and butyl hexanoate, whereas variables such as limonene and methyl salicylate showed stronger responses in external-origin samples. The coexistence of WZ-high and non-WZ-high variables indicates that the discriminant information depends on the relative response configuration of multiple VOC signals rather than on the response of any single VOC signal variable.

For juice samples, 26 VOC signal variables satisfied the same combined criterion. The selected variables also represented multiple chemical classes and included both WZ-high and non-WZ-high response patterns. Representative WZ-associated signals included beta-ocimene, 2-acetylfuran, furfuryl alcohol, pentanoic acid, and limonene, whereas several ester-related and aldehyde-related variables showed stronger responses in external-origin samples. Compared with peel, the discriminant information in juice was distributed across a broader range of VOC signals, which was consistent with the more dispersed fingerprint pattern observed in the Gallery Plot and PCA results.

Overall, WZ-origin Ougan could be distinguished from non-WZ samples by integrating multiple discriminant VOC signal variables rather than by relying on a single compound. Peel samples showed more WZ-high discriminant variables and clearer WZ-related fingerprint characteristics, whereas juice samples provided additional model-based discrimination information. Therefore, the identification of Wenzhou-origin Ougan should be based on the overall VOC fingerprint pattern formed by both WZ-high and non-WZ-high signals.

### 3.5. Comparison of Peel and Juice for WZ-Origin Discrimination

The above results indicate that peel and juice samples provided different types of information for the identification of Wenzhou-origin Ougan. Peel samples showed more concentrated fingerprint differences in the Gallery Plot, two-dimensional GC-IMS spectra and PCA score plot. The WZ peel samples were more visually distinguishable from samples of external origins, suggesting that peel VOC fingerprints are more suitable for intuitive comparison and interpretation of WZ-related fingerprint characteristics.

In contrast, juice samples showed more dispersed VOC response patterns. Although the visual separation of juice samples was less concentrated than that of peel samples, the juice PLS-DA model showed a slightly higher Q^2^ value than the peel model. This suggests that juice samples contained useful multivariable information for model-based origin discrimination. Therefore, the role of juice samples should not be evaluated only by visual fingerprint concentration; their contribution is more reflected in supervised multivariate classification.

From the perspective of discriminant VOC signal variables, peel samples contained more variables satisfying VIP > 1 and *p* < 0.05 than juice samples. In addition, peel samples contained more WZ-high variables, indicating that peel VOC fingerprints provided clearer WZ-origin response characteristics. Juice samples contained fewer WZ-high variables, but their discriminant variables were distributed across more chemical classes and origin-response types, indicating a broader multivariable discrimination pattern. From the perspective of WZ-origin identification, the score plots and the selected discriminant VOC signal variables showed that WZ samples had distinguishable fingerprint characteristics compared with samples from external origins. In other words, the model results support the feasibility of identifying Wenzhou-origin Ougan within a four-origin classification framework, while possible misclassification among external origins should be interpreted separately from the recognition of WZ samples.

Taken together, peel and juice have different advantages and limitations as matrices for geographical origin discrimination. Peel produced more concentrated fingerprint differences, more WZ-high discriminant variables, and clearer visual separation, and only a small amount of peel tissue was required in the present HS-GC-IMS procedure. These characteristics make peel potentially more convenient for rapid fingerprint-based screening and interpretation. However, peel volatiles may be more directly influenced by surface exposure, handling, and peel-specific physiological conditions, which should be considered when applying the method to samples collected under different postharvest conditions. Juice, in contrast, showed more dispersed VOC patterns but provided useful multivariable information and a slightly higher Q^2^ value in the current PLS-DA model. Its limitation is that sample preparation requires juice extraction and homogenization, and the resulting VOC profile may also be affected by pulp composition and fruit maturity. Therefore, based on the present results, peel may be considered the more practical primary matrix for routine rapid screening of Wenzhou Ougan origin, whereas juice is better regarded as a complementary matrix that can provide additional model-based discrimination information. This interpretation should nevertheless be validated using larger and independent sample sets before routine authentication is established.

From a practical perspective, HS-GC-IMS provides a relatively rapid fingerprinting workflow with simple headspace sample preparation and visually interpretable multidimensional VOC information, which supports its potential use as a screening tool for geographical origin authentication. However, routine application would require further standardization of sample collection and preparation, establishment of a sufficiently representative reference fingerprint database, and validation of classification performance using independent samples collected across different harvest years and production batches. In addition, the discriminant variables screened in this study should be regarded as tentatively annotated VOC signal variables rather than chemically confirmed marker compounds, because their assignments were based on retention index and drift time matching with the GC-IMS library. Complementary GC-MS analysis, preferably together with authentic standards, would provide higher confidence in the chemical identities of the most important discriminant signals [21,22]. Therefore, the present HS-GC-IMS approach is more appropriately regarded as a promising rapid fingerprint-screening strategy rather than a fully validated routine authentication method.

### 3.6. Limitations and Future Perspectives

Several limitations of this study should be acknowledged. First, the sample sizes were relatively limited and unbalanced among geographical groups, with more samples collected from Wenzhou than from the three external origins. This imbalance may affect the representativeness and generalizability of the classification results. Second, all samples were collected during a single harvest period, and the stability of the observed VOC fingerprint patterns across different harvest years has not yet been evaluated. Third, although fruits with similar maturity were selected and the harvest dates were kept as similar as possible, environmental conditions, cultivation practices, maturity, and postharvest factors were not independently controlled and may have contributed to the observed origin-related VOC differences. Fourth, no completely independent external sample set was available for model validation, and the current results should therefore be regarded as preliminary evidence of geographical discrimination rather than as validation of a routine authentication model. Finally, the compound assignments were tentative annotations based on RI and DT matching with the GC-IMS library and were not confirmed using complementary GC-MS analysis or authentic standards. Future studies should therefore include larger and more balanced sample sets, samples collected across multiple harvest years and production batches, independent external validation, and complementary chemical confirmation to further evaluate the robustness and applicability of the proposed HS-GC-IMS fingerprinting approach. Future studies could also investigate the integration of standardized image-based features with HS-GC-IMS fingerprints to determine whether visual and volatile information provide complementary value for geographical-origin discrimination, particularly across different maturity stages and postharvest periods.

## 4. Conclusions

In this study, HS-GC-IMS combined with chemometric analysis was used to characterize VOC fingerprints of Ougan mandarin peel and juice samples from WZ, GX, FJ and SC. The peel and juice VOC fingerprints were represented by 59 and 68 VOC signal variables, respectively, which were classified into nine chemical classes, including esters, alcohols, aldehydes, ketones, acids, terpenes and terpenoids, furans and furan derivatives, aromatic compounds, and others. The GC-IMS Gallery Plot and two-dimensional spectra showed visible origin-related differences in both peel and juice samples, indicating that VOC fingerprints could provide useful information for geographical origin discrimination.

PCA showed that both peel and juice samples contained origin-related variation in their VOC fingerprints. Peel samples exhibited more concentrated differential regions and clearer visual separation between WZ and non-WZ samples, suggesting that peel fingerprints were more suitable for intuitive interpretation of Wenzhou-origin characteristics. PLS-DA further supported the feasibility of origin discrimination. The peel model achieved an R^2^Y of 0.710 and a Q^2^ of 0.678, while the juice model achieved an R^2^Y of 0.707 and a Q^2^ of 0.692. Permutation tests indicated that the discrimination was not mainly caused by random class assignment.

By combining VIP values from the PLS-DA models with Welch’s ANOVA results, 30 peel variables and 26 juice variables satisfied the criterion of VIP > 1 and *p* < 0.05. These variables were screened as discriminant VOC signal variables for WZ-origin discrimination. In peel samples, 14 selected variables showed the highest responses in WZ samples, including furfuryl alcohol, 3-octanone, pentanal, n-nonanal, ethyl 2-methylbutyrate, butyl hexanoate, alpha-pinene, 3-methylbutyric acid, benzyl alcohol, decanal, 2-ethylfuran, 2-heptanone, styrene and pentanoic acid. In juice samples, 10 selected variables showed the highest responses in WZ samples, including styrene, pentanoic acid, beta-ocimene, 2-acetylfuran, furfuryl alcohol, butyric acid, benzyl alcohol, 2-methylfuran, ethanol and limonene. These WZ-high signals, together with non-WZ-high variables, formed the multivariable fingerprint pattern for distinguishing Wenzhou-origin Ougan from external-origin samples.

Overall, the geographical origin discrimination of Wenzhou Ougan in the present study was supported by an integrated VOC fingerprint pattern rather than by a single VOC signal variable. Peel and juice samples provided complementary information: peel showed clearer visual fingerprint characteristics, whereas juice provided additional multivariable information for model-based discrimination. These findings demonstrate considerable potential for the use of HS-GC-IMS as a rapid fingerprinting approach for geographical discrimination of Wenzhou Ougan. However, further validation using larger and independent sample sets from different harvest periods, together with complementary analytical techniques such as GC-MS and authentic standards for chemical confirmation, is required before the approach can be reliably applied to routine origin authentication.

## Figures and Tables

**Table 1:** Sample sources and code description.

Sample Type	Geographical Origins Code	Number of Samples Per Group	Total Number of Samples
Juice	WZ1–WZ4, GX, FJ, SC	WZ 30, GX 6, FJ 6, SC 5	47
Peel	WZ1–WZ4, GX, FJ, SC	WZ 16, GX 6, FJ 6, SC 6	34

**Table 2:** Tentatively annotated VOC signal variables and relative response values of Ougan peel samples from different origins.

Tentative Annotation	RI	RT (s)	DT (ms)	WZ	GX	FJ	SC
Esters				659.06 ± 42.11 a	754.67 ± 72.89 a	682.00 ± 84.18 a	686.83 ± 41.63 a
ethyl acetate	597.900	186.913	1.077	44.88 ± 4.98 c	41.17 ± 4.58 c	66.67 ± 9.79 b	87.50 ± 11.50 a
iso-Propyl acetate	637.900	208.158	1.142	93.56 ± 1.09 b	99.17 ± 0.41 a	81.83 ± 3.66 c	86.33 ± 7.97 bc
ethyl propanoate	644.500	211.913	1.462	38.00 ± 35.05 a	34.67 ± 24.40 a	13.17 ± 2.93 a	19.33 ± 5.89 a
methyl 2-methylpropanoate	697.600	245.553	1.532	13.38 ± 17.85 a	4.17 ± 3.06 a	37.67 ± 45.60 a	4.17 ± 1.47 a
n-Propyl acetate	698.100	245.902	1.482	27.62 ± 28.11 a	16.67 ± 13.40 ab	9.17 ± 3.82 ab	6.00 ± 1.41 b
ethyl propanoate	733.800	272.081	1.447	36.62 ± 12.58 c	22.50 ± 8.17 d	92.50 ± 4.64 a	84.67 ± 3.83 b
ethyl 2-methylpropanoate	737.000	274.593	1.557	75.31 ± 14.01 b	94.50 ± 4.72 a	7.33 ± 0.52 c	5.00 ± 0.63 d
2-Methyl propyl acetate	750.200	285.182	1.608	13.19 ± 3.47 a	38.67 ± 39.09 abc	7.00 ± 2.19 b	2.00 ± 2.00 c
ethyl 2-methylbutyrate	836.100	366.902	1.643	91.00 ± 4.18 a	87.17 ± 8.04 a	66.33 ± 7.06 b	65.17 ± 4.45 b
butyl propanoate	896.700	440.129	1.292	53.69 ± 13.15 a	75.67 ± 19.87 a	47.67 ± 25.86 a	54.83 ± 11.03 a
(Z)-3-Hexenyl acetate	1003.400	610.808	1.809	33.19 ± 6.34 b	85.67 ± 16.48 a	41.83 ± 7.78 b	16.17 ± 4.02 c
methyl salicylate	1131.800	913.976	1.217	18.94 ± 6.60 b	22.33 ± 7.58 b	69.83 ± 7.81 a	78.83 ± 15.82 a
butyl hexanoate	1161.900	1004.887	1.448	56.62 ± 24.13 a	1.67 ± 0.52 b	1.83 ± 0.41 b	2.83 ± 2.04 b
Ethyl benzoate	1202.900	1143.715	1.725	54.00 ± 27.46 c	91.33 ± 3.33 a	78.33 ± 2.34 b	87.83 ± 8.47 ab
ethyl octanoate	1213.300	1181.563	1.480	9.06 ± 4.57 c	39.33 ± 19.19 b	60.83 ± 9.20 b	86.17 ± 13.32 a
Alcohols				700.00 ± 101.53 b	835.83 ± 81.98 a	653.33 ± 30.97 b	601.00 ± 23.01 c
ethanol	415.300	118.858	1.133	80.50 ± 10.83 a	82.50 ± 3.39 a	66.00 ± 7.13 b	83.33 ± 6.98 a
Isopropyl alcohol	490.300	141.951	1.170	43.50 ± 32.41 ab	31.33 ± 19.94 bc	71.67 ± 21.95 a	9.33 ± 11.50 c
1-butanol	644.200	211.761	1.185	75.12 ± 12.47 b	93.33 ± 5.05 a	16.50 ± 12.42 c	9.33 ± 2.58 c
3-methylbutan-1-ol	692.100	241.791	1.254	73.94 ± 19.82 a	88.33 ± 6.89 a	18.00 ± 3.63 b	18.50 ± 2.07 b
2,3-Butanediol	789.300	319.540	1.363	43.12 ± 17.72 b	82.83 ± 20.91 a	19.67 ± 10.13 c	78.50 ± 12.72 a
2-Hexanol	799.800	329.491	1.585	39.31 ± 27.61 c	59.50 ± 15.73 bc	72.33 ± 8.82 b	96.83 ± 1.83 a
2-methylpentan-1-ol	823.400	353.283	2.178	65.75 ± 9.69 a	66.67 ± 7.26 a	46.00 ± 5.73 b	43.33 ± 9.46 b
3-Methyl-1-pentanol	850.800	383.297	1.301	7.75 ± 2.65 b	61.00 ± 34.89 a	78.33 ± 11.79 a	55.83 ± 15.04 a
octan-3-ol	954.100	524.503	1.788	36.81 ± 8.37 a	57.67 ± 24.48 a	12.67 ± 4.08 b	12.00 ± 3.58 b
benzyl alcohol	1017.400	638.032	1.514	90.88 ± 5.32 a	53.83 ± 19.35 b	61.33 ± 17.49 b	60.83 ± 8.42 b
1-Octanol	1043.600	692.583	1.458	69.50 ± 6.57 b	69.33 ± 27.08 abc	84.17 ± 9.30 a	57.33 ± 6.62 c
(Z)-3-octen-1-ol	1084.200	786.660	1.726	61.50 ± 25.54 a	12.50 ± 3.89 b	52.83 ± 20.59 a	42.00 ± 12.88 a
2-Phenylethanol	1109.600	852.245	1.306	12.31 ± 2.52 c	77.00 ± 26.00 a	53.83 ± 9.09 a	33.83 ± 6.08 b
Aldehydes				371.88 ± 17.87 a	274.17 ± 18.51 b	300.33 ± 36.83 b	302.67 ± 27.95 b
butanal	545.100	162.849	1.083	73.81 ± 2.66 b	80.83 ± 2.79 a	81.17 ± 7.19 ab	90.50 ± 10.75 a
pentanal	685.600	237.443	1.427	92.38 ± 7.56 a	65.67 ± 19.99 a	17.00 ± 22.10 b	1.33 ± 0.52 b
hexanal	762.000	295.119	1.259	9.56 ± 6.13 c	36.50 ± 9.14 b	75.83 ± 18.28 a	60.83 ± 17.57 ab
propanal	778.700	309.790	1.044	59.38 ± 10.61 c	70.50 ± 27.14 abc	78.33 ± 8.91 b	93.50 ± 6.72 a
n-nonanal	1130.400	910.012	1.451	61.25 ± 21.29 a	2.83 ± 0.41 b	1.67 ± 0.52 c	1.83 ± 0.41 c
decanal	1153.600	979.182	2.045	75.50 ± 16.40 a	17.83 ± 18.35 c	46.33 ± 7.20 b	54.67 ± 11.20 b
Ketones				566.75 ± 26.86 b	477.00 ± 22.16 c	499.00 ± 34.81 c	660.67 ± 47.25 a
2-Propanone	508.200	148.391	1.114	73.81 ± 16.53 b	89.67 ± 5.85 a	54.00 ± 29.63 ab	94.17 ± 8.57 a
2-butanone	600.600	188.236	1.241	27.19 ± 4.67 b	32.00 ± 5.51 b	35.00 ± 14.00 b	79.67 ± 15.40 a
3-Pentanone	670.200	227.464	1.351	35.69 ± 11.70 b	91.67 ± 9.44 a	41.00 ± 28.90 b	84.50 ± 10.21 a
2-pentanone	696.200	244.589	1.129	57.75 ± 11.53 b	73.83 ± 13.99 ab	53.00 ± 22.77 ab	80.83 ± 10.65 a
2,3-pentanedione	702.500	248.921	1.212	50.12 ± 2.75 a	45.67 ± 3.67 a	51.67 ± 35.99 ab	34.00 ± 5.48 b
2-heptanone	896.900	440.387	1.651	79.25 ± 10.77 a	46.17 ± 8.08 c	68.17 ± 2.64 b	49.67 ± 10.07 c
3-octanone	968.500	548.290	1.696	94.62 ± 2.85 a	18.50 ± 12.71 c	76.83 ± 6.01 b	82.67 ± 8.02 b
butane-2,3-dione	1002.700	609.457	1.181	95.50 ± 1.93 ab	10.33 ± 3.39 c	97.50 ± 1.87 a	93.50 ± 2.07 b
Acetophenone	1050.100	706.858	1.172	52.81 ± 11.50 a	69.17 ± 20.70 a	21.83 ± 4.79 b	61.67 ± 18.80 a
Acids				335.50 ± 19.44 a	310.17 ± 26.50 a	308.67 ± 23.97 a	328.17 ± 28.91 a
butyric acid	747.400	282.928	1.398	58.62 ± 20.29 a	80.67 ± 19.83 a	53.17 ± 7.86 a	61.17 ± 8.35 a
3-methylbutyric acid	845.900	377.762	1.214	88.19 ± 5.61 a	54.17 ± 21.03 bc	38.83 ± 3.97 c	57.33 ± 10.01 b
2-methylbutyric acid	858.000	391.691	1.460	21.50 ± 8.73 d	84.00 ± 8.46 a	64.67 ± 6.92 b	43.67 ± 3.88 c
pentanoic acid	946.400	512.254	1.513	81.75 ± 8.97 a	24.00 ± 15.96 b	58.33 ± 19.86 a	77.67 ± 30.47 a
Hexanoic acid	990.600	587.086	1.654	85.44 ± 7.31 b	67.33 ± 10.15 c	93.67 ± 4.41 a	88.33 ± 4.76 ab
Terpenes and terpenoids				338.38 ± 35.29 b	275.50 ± 53.25 b	378.00 ± 20.61 a	387.17 ± 30.82 a
alpha-pinene	904.500	450.629	1.206	82.88 ± 11.41 a	26.67 ± 14.94 c	65.67 ± 7.23 b	56.50 ± 11.73 b
limonene	1051.900	710.800	1.720	14.62 ± 3.16 d	89.67 ± 6.09 a	73.83 ± 5.42 b	47.50 ± 9.44 c
Linalool	1068.000	747.732	1.692	84.12 ± 6.22 a	63.83 ± 17.06 a	67.67 ± 20.34 a	85.33 ± 5.28 a
1-Menthol	1136.800	928.681	1.891	4.06 ± 1.95 c	15.50 ± 5.21 b	53.00 ± 5.73 a	52.83 ± 24.59 a
(E)-3,7-Dimethyl-2,6-octadien-1-ol	1212.400	1178.344	1.218	86.12 ± 9.09 a	62.17 ± 20.70 ab	68.50 ± 6.12 b	74.33 ± 3.83 b
(E)-3,7-Dimethyl-2,6-octadien-1-ol	1319.200	1649.899	1.221	66.56 ± 27.84 a	17.67 ± 5.99 b	49.33 ± 5.89 a	70.67 ± 17.47 a
Furans and furan derivatives				208.19 ± 39.05 a	125.00 ± 32.03 b	127.17 ± 32.70 b	106.33 ± 6.98 b
2-ethylfuran	700.100	247.309	1.299	84.81 ± 9.76 a	57.83 ± 3.06 b	64.67 ± 12.80 b	65.83 ± 5.42 b
furfuryl alcohol	845.000	376.669	1.108	74.06 ± 12.16 a	14.50 ± 4.51 b	19.67 ± 4.55 b	20.17 ± 2.56 b
furfuryl alcohol	858.700	392.519	1.383	49.31 ± 36.81 a	52.67 ± 31.26 ab	42.83 ± 19.43 ab	20.33 ± 4.46 b
Aromatic compounds				130.25 ± 19.00 a	47.33 ± 25.76 b	78.67 ± 8.19 b	107.67 ± 16.91 a
styrene	850.700	383.197	1.045	59.38 ± 14.88 a	26.83 ± 17.79 bc	23.50 ± 3.27 c	32.17 ± 5.56 b
Eugenol	1320.100	1654.502	1.302	70.88 ± 27.98 ab	20.50 ± 8.12 c	55.17 ± 6.59 b	75.50 ± 12.34 a

Note: Data are expressed as mean ± SD. Different lowercase letters in the same row indicate significant differences among geographical origins based on Welch’s one-way ANOVA followed by the Games–Howell post hoc test (*p* < 0.05). RI, retention index; RT, retention time; DT, drift time. WZ, GX, FJ and SC represent Wenzhou, Guangxi, Fujian and Sichuan, respectively. The listed variables are VOC signal variables used for subsequent statistical analysis. The response values represent normalized GC-IMS peak-volume intensities rather than absolute concentrations.

**Table 3:** Tentatively annotated VOC signal variables and relative response values of Ougan juice samples from different origins.

Tentative Annotation	RI	RT (s)	DT (ms)	WZ	GX	FJ	SC
Esters				497.43 ± 77.82 b	929.50 ± 42.02 a	488.00 ± 43.65 bc	417.60 ± 34.85 c
ethyl acetate	582.600	179.477	1.334	85.17 ± 11.19 b	94.83 ± 4.12 a	24.83 ± 13.44 c	14.60 ± 0.89 c
iso-Propyl acetate	653.100	216.997	1.503	16.40 ± 17.15 b	84.33 ± 16.77 a	5.00 ± 4.56 c	1.00 ± 0.00 c
ethyl propanoate	697.000	245.155	1.134	75.33 ± 21.22 a	60.50 ± 3.62 b	64.17 ± 37.74 abc	26.40 ± 10.60 c
2-Methyl propyl acetate	751.500	286.296	1.602	10.80 ± 2.92 b	87.33 ± 15.16 a	8.83 ± 2.14 b	1.20 ± 0.45 c
ethyl butyrate	763.700	296.603	1.805	41.43 ± 13.77 a	18.50 ± 8.55 b	55.50 ± 30.53 ab	27.80 ± 19.69 ab
ethyl 2-methylpropanoate	771.500	303.366	1.212	56.00 ± 25.25 b	88.50 ± 9.16 a	4.33 ± 3.14 c	2.40 ± 0.55 c
ethybutanl 2-methylbutyrate	823.100	352.965	1.645	29.33 ± 22.19 c	53.83 ± 10.61 b	78.67 ± 17.78 ab	90.00 ± 8.28 a
3-methylbutyl acetate	828.500	358.676	1.736	35.57 ± 31.83 b	71.50 ± 5.09 a	70.33 ± 6.09 a	46.00 ± 13.64 b
butyl propanoate	873.900	410.854	1.293	82.43 ± 12.91 a	61.50 ± 6.53 b	79.00 ± 6.42 a	86.80 ± 3.70 a
Butyl butyrate	979.000	566.326	1.344	25.73 ± 16.92 b	85.67 ± 11.09 a	14.17 ± 2.04 c	23.60 ± 1.34 b
n-hexyl acetate	1016.500	636.266	1.419	32.50 ± 19.43 d	50.00 ± 6.99 c	76.00 ± 9.49 b	93.80 ± 6.87 a
Isopentyl 2-methyl butanoate	1115.100	867.096	2.025	1.00 ± 0.37 b	80.33 ± 18.29 a	1.17 ± 0.41 b	1.00 ± 0.00 b
butyl hexanoate	1162.000	1005.314	1.448	5.73 ± 3.20 b	92.67 ± 4.08 a	6.00 ± 2.28 bc	3.00 ± 0.71 c
Alcohols				499.40 ± 91.22 b	600.00 ± 15.58 a	387.00 ± 30.05 c	356.00 ± 17.90 c
ethanol	416.700	119.242	1.131	73.37 ± 18.00 a	49.00 ± 15.40 b	34.17 ± 16.63 b	42.60 ± 7.96 b
1-Propanol	525.400	154.901	1.112	96.07 ± 2.59 b	90.17 ± 6.01 ab	96.33 ± 1.37 ab	98.00 ± 1.00 a
2-Methyl-1-propanol	603.000	189.480	1.369	56.83 ± 25.16 a	54.17 ± 7.00 a	12.50 ± 3.15 b	14.20 ± 1.64 b
3-methylbutan-2-ol	652.100	216.364	1.446	37.47 ± 27.29 b	88.50 ± 6.89 a	15.00 ± 13.21 c	0.00 ± 0.00 c
3-methylbutan-1-ol	741.200	277.933	1.224	54.57 ± 19.34 b	20.33 ± 5.85 c	79.00 ± 5.25 a	53.80 ± 6.57 b
2-Hexanol	786.300	316.759	1.559	37.97 ± 22.16 c	54.67 ± 2.42 b	41.17 ± 6.46 c	88.60 ± 7.83 a
2-Methyl-1-butanol	798.000	327.747	1.469	41.77 ± 28.94 a	33.50 ± 6.80 a	6.67 ± 1.21 b	7.20 ± 1.30 b
2-methylpentan-1-ol	803.100	332.749	1.301	34.30 ± 21.06 b	18.83 ± 2.56 c	93.50 ± 4.09 a	30.60 ± 6.43 b
(E)-3-hexen-1-ol	844.700	376.344	1.542	2.77 ± 1.04 c	88.33 ± 10.46 a	2.67 ± 0.82 c	7.60 ± 1.82 b
octan-3-ol	959.600	533.400	1.792	12.23 ± 7.66 b	96.33 ± 3.01 a	1.50 ± 1.22 c	0.60 ± 0.55 c
benzyl alcohol	1015.700	634.652	1.330	52.07 ± 27.89 a	6.17 ± 4.88 bc	4.50 ± 2.88 c	12.80 ± 2.59 b
Aldehydes				560.20 ± 95.20 b	720.83 ± 19.32 a	677.33 ± 81.18 abc	644.20 ± 42.79 c
propanal	488.300	141.281	1.065	57.47 ± 24.28 a	40.83 ± 3.25 b	29.83 ± 17.30 b	50.20 ± 9.36 ab
butanal	586.900	181.565	1.286	75.13 ± 11.60 a	80.00 ± 22.03 a	43.33 ± 23.04 ab	20.60 ± 3.13 b
pentanal	685.600	237.436	1.428	58.13 ± 25.63 a	54.17 ± 17.47 ab	39.83 ± 7.31 b	8.60 ± 0.55 c
2-methyl-2-butenal	716.500	258.986	1.664	58.07 ± 26.69 c	95.50 ± 3.73 a	82.00 ± 10.45 ab	77.60 ± 8.88 b
3-methylbut-2-enal	736.300	274.038	1.688	30.20 ± 7.29 a	1.67 ± 1.21 b	65.17 ± 26.18 a	9.20 ± 5.07 b
3-methylbut-2-enal	752.800	287.352	1.645	13.23 ± 14.57 b	67.33 ± 27.30 a	3.00 ± 1.10 c	1.40 ± 0.55 c
Heptanal	924.100	478.373	1.355	67.13 ± 23.70 a	39.00 ± 8.94 b	44.50 ± 5.01 b	34.40 ± 10.60 b
2-heptenal (E)	954.800	525.658	1.247	36.10 ± 8.70 b	5.00 ± 0.00 c	34.83 ± 3.66 b	79.40 ± 12.95 a
octanal	1014.900	633.148	1.825	5.33 ± 7.84 b	55.50 ± 7.97 a	79.33 ± 18.41 a	52.00 ± 18.68 a
2,4-heptadienal (E,E)	1018.000	639.223	1.207	50.60 ± 23.41 b	31.50 ± 3.39 c	76.33 ± 9.50 a	42.00 ± 9.67 bc
(E)-oct-2-enal	1056.400	720.796	1.814	35.50 ± 19.75 c	61.17 ± 2.79 b	39.00 ± 7.27 c	86.80 ± 8.56 a
(E)-oct-2-enal	1105.300	840.731	1.330	21.67 ± 9.39 c	26.33 ± 6.74 c	50.67 ± 11.94 b	86.00 ± 8.09 a
n-nonanal	1114.400	865.254	1.485	11.80 ± 6.33 b	93.67 ± 3.39 a	8.83 ± 6.08 bc	4.80 ± 1.10 c
decanal	1154.600	982.267	1.554	39.83 ± 20.67 c	69.17 ± 4.31 b	80.67 ± 5.82 a	91.20 ± 9.07 a
Ketones				506.73 ± 58.71 b	400.00 ± 19.57 c	537.67 ± 15.58 b	764.80 ± 14.65 a
butane-2,3-dione	533.200	157.986	1.155	45.47 ± 17.40 b	77.00 ± 21.19 a	9.33 ± 3.39 c	15.00 ± 6.08 c
2-butanone	591.300	183.682	1.070	51.63 ± 11.08 b	7.83 ± 5.64 c	63.00 ± 20.45 b	93.60 ± 3.65 a
1-Hydroxy-2-propanone	663.700	223.427	1.044	22.00 ± 10.04 c	16.17 ± 6.85 c	58.67 ± 15.10 b	85.40 ± 11.24 a
3-Pentanone	673.600	229.611	1.351	48.10 ± 29.24 b	87.33 ± 13.46 a	28.00 ± 8.07 c	83.40 ± 12.32 a
2,3-pentanedione	707.600	252.584	1.301	38.20 ± 17.65 b	52.83 ± 9.99 b	82.33 ± 9.91 a	89.40 ± 9.91 a
2-pentanone	722.500	263.442	1.364	21.90 ± 27.84 a	5.33 ± 2.16 b	3.17 ± 0.75 b	4.60 ± 0.89 b
acetoin (3-hydroxy-2-butanone)	723.000	263.862	1.078	35.57 ± 11.25 b	10.67 ± 3.27 c	18.50 ± 7.23 c	82.20 ± 12.93 a
2-heptanone	890.100	431.402	1.709	34.53 ± 29.60 b	34.00 ± 3.95 b	91.00 ± 5.06 a	26.80 ± 5.40 b
2-Octanone	961.800	537.033	1.344	45.87 ± 24.76 a	29.50 ± 2.51 b	17.33 ± 8.33 b	4.40 ± 0.55 c
3-octanone	964.700	541.853	1.697	71.20 ± 15.94 b	19.50 ± 2.17 d	59.50 ± 4.64 c	97.00 ± 3.00 a
6-Methyl-5-hepten-2-one	1004.600	613.185	1.184	66.87 ± 9.08 b	41.00 ± 2.97 c	69.50 ± 14.27 b	91.00 ± 5.10 a
Acetophenone	1051.900	710.747	1.175	25.40 ± 5.81 c	18.83 ± 4.45 c	37.33 ± 7.55 b	92.00 ± 6.89 a
Acids				379.43 ± 58.22 a	334.83 ± 34.13 ab	281.00 ± 26.80 bc	253.40 ± 14.45 c
Acetic acid	551.200	165.411	1.048	77.07 ± 17.09 ab	88.50 ± 8.50 a	55.17 ± 15.85 bc	52.40 ± 4.04 c
Propanoic acid	714.500	257.568	1.267	41.80 ± 26.46 a	22.83 ± 4.45 b	21.33 ± 7.17 bc	11.80 ± 2.59 c
butyric acid	797.600	327.358	1.375	56.60 ± 20.23 a	28.83 ± 7.88 b	19.33 ± 10.01 bc	9.00 ± 2.45 c
2-methylbutyric acid	818.600	348.320	1.213	67.53 ± 18.40 a	70.33 ± 8.64 ab	83.00 ± 11.61 ab	81.40 ± 1.52 b
2-methylbutyric acid	871.800	408.212	1.213	67.93 ± 17.68 c	87.33 ± 10.01 ab	91.50 ± 3.99 a	74.80 ± 8.76 bc
pentanoic acid	929.300	486.014	1.501	68.50 ± 14.09 a	37.00 ± 11.24 b	10.67 ± 8.09 c	24.00 ± 3.94 b
Terpenes and terpenoids				200.30 ± 31.05 a	154.67 ± 10.21 b	136.00 ± 46.00 abc	98.60 ± 7.67 c
isoprene	484.700	140.041	1.196	79.23 ± 21.79 a	67.33 ± 5.09 a	45.50 ± 24.68 ab	43.80 ± 5.63 b
alpha-pinene	937.200	497.914	1.679	9.80 ± 6.17 c	64.00 ± 14.27 a	67.00 ± 23.86 a	27.00 ± 4.30 b
limonene	1016.400	636.088	1.230	52.00 ± 24.55 a	16.00 ± 4.05 b	14.67 ± 4.08 b	14.00 ± 2.00 b
beta-ocimene	1044.900	695.253	1.212	59.27 ± 23.68 a	7.33 ± 1.03 c	8.83 ± 3.06 bc	13.80 ± 3.11 b
Furans and furan derivatives				268.40 ± 29.81 a	130.00 ± 9.82 c	196.00 ± 29.41 b	187.00 ± 12.94 b
2-methylfuran	602.500	189.201	1.403	65.63 ± 24.30 a	8.00 ± 2.10 c	38.67 ± 8.96 b	38.00 ± 8.46 b
2-ethylfuran	700.100	247.310	1.044	30.07 ± 19.18 c	5.00 ± 2.00 d	81.33 ± 16.15 a	48.20 ± 2.77 b
furfuryl alcohol	835.700	366.367	1.130	58.50 ± 23.33 a	26.67 ± 10.48 b	13.00 ± 5.18 bc	8.80 ± 0.84 c
2-acetylfuran	898.600	442.623	1.440	47.87 ± 22.06 a	6.33 ± 2.07 b	9.50 ± 1.97 b	8.60 ± 3.13 b
2-Pentylfuran	979.800	567.672	1.247	66.33 ± 5.60 b	84.00 ± 8.02 a	53.50 ± 5.96 c	83.40 ± 13.13 ab
Aromatic compounds				122.50 ± 32.06 a	73.83 ± 23.94 b	27.33 ± 8.85 c	88.20 ± 17.96 b
styrene	889.400	430.543	1.038	77.60 ± 15.16 a	40.83 ± 9.33 b	13.17 ± 6.85 c	21.60 ± 4.28 c
ortho-Guaiacol	1081.500	780.008	1.250	44.90 ± 25.90 a	33.00 ± 16.69 ab	14.17 ± 4.45 b	66.60 ± 21.78 a
Others				59.40 ± 16.15 a	14.33 ± 2.42 b	41.00 ± 17.62 a	71.00 ± 19.57 a
diethoxy-1,1-ethane	753.400	287.842	1.043	59.40 ± 16.15 a	14.33 ± 2.42 b	41.00 ± 17.62 a	71.00 ± 19.57 a

Note: Data are expressed as mean ± SD. Different lowercase letters in the same row indicate significant differences among geographical origins based on Welch’s one-way ANOVA followed by the Games–Howell post hoc test (*p* < 0.05). RI, retention index; RT, retention time; DT, drift time. WZ, GX, FJ and SC represent Wenzhou, Guangxi, Fujian and Sichuan, respectively. The listed variables are VOC signal variables used for subsequent statistical analysis. The response values represent normalized GC-IMS peak-volume intensities rather than absolute concentrations.

**Table 4:** Comparison of PCA/PLS-DA model performance and screened discriminant VOC signal variables between peel and juice samples.

Sample Type	Number of VOC Signal Variables	PC1/%	PC2/%	PC1 + PC2/%	R^2^Y	Q^2^	Number of Variables with VIP > 1	Number of Variables with VIP > 1 and *p* < 0.05
Peel	59	35.68	22.64	58.33	0.710	0.678	33	30
Juice	68	32.67	24.42	57.09	0.707	0.692	29	26

**Table 5:** Cross-validated classification accuracy of different algorithms for peel and juice VOC fingerprints.

Sample Type	PLS-DA	SVM	Random Forest
Peel	0.790 ± 0.068	0.983 ± 0.069	0.997 ± 0.020
Juice	0.873 ± 0.037	1.000 ± 0.000	1.000 ± 0.000

Note: Values are expressed as mean ± SD from repeated stratified five-fold cross-validation with 10 repeats. All three classifiers were evaluated using identical cross-validation partitions.

**Table 6:** Discriminant VOC signal variables for WZ-origin discrimination selected by VIP > 1 and *p* < 0.05.

No.	Key Discriminant VOC Signal Variables in Peel	Peel VIP	High-Response Origin in Peel	Key Discriminant VOC Signal Variables in Juice	Juice VIP	High-Response Origin in Juice
1	furfuryl alcohol	1.330	WZ	octanal	1.538	FJ
2	limonene	1.317	GX	styrene	1.483	WZ
3	3-octanone	1.297	WZ	pentanoic acid	1.441	WZ
4	2-methylbutyric acid	1.276	GX	alpha-pinene	1.416	FJ
5	pentanal	1.271	WZ	beta-ocimene	1.332	WZ
6	ethyl octanoate	1.254	SC	ethyl acetate	1.272	GX
7	n-nonanal	1.252	WZ	butyl hexanoate	1.266	GX
8	methyl salicylate	1.249	SC	decanal	1.262	SC
9	ethyl 2-methylpropanoate	1.249	GX	Isopentyl 2-methyl butanoate	1.256	GX
10	1-butanol	1.235	GX	n-hexyl acetate	1.255	SC
11	hexanal	1.230	FJ	2-acetylfuran	1.250	WZ
12	2-Phenylethanol	1.220	GX	ethyl 2-methylbutyrate	1.236	SC
13	1-Menthol	1.217	FJ	furfuryl alcohol	1.231	WZ
14	ethyl propanoate	1.215	FJ	butyric acid	1.230	WZ
15	ethyl 2-methylbutyrate	1.210	WZ	n-nonanal	1.227	GX
16	butyl hexanoate	1.196	WZ	2-Methyl propyl acetate	1.206	GX
17	alpha-pinene	1.195	WZ	1-Hydroxy-2-propanone	1.197	SC
18	3-Methyl-1-pentanol	1.191	FJ	octan-3-ol	1.180	GX
19	3-methylbutyric acid	1.186	WZ	benzyl alcohol	1.177	WZ
20	3-methylbutan-1-ol	1.170	GX	2-methylfuran	1.171	WZ
21	benzyl alcohol	1.149	WZ	ethanol	1.149	WZ
22	ethyl acetate	1.141	SC	limonene	1.144	WZ
23	decanal	1.137	WZ	butanal	1.063	GX
24	2-ethylfuran	1.110	WZ	Butyl butyrate	1.034	GX
25	2-heptanone	1.090	WZ	iso-Propyl acetate	1.021	GX
26	styrene	1.088	WZ	Acetophenone	1.004	SC
27	iso-Propyl acetate	1.059	GX			
28	octan-3-ol	1.052	GX			
29	pentanoic acid	1.031	WZ			
30	3-Pentanone	1.009	GX			

Note: Discriminant VOC signal variables listed in this table were selected using the combined criterion of VIP > 1 and *p* < 0.05. VIP values were calculated from the PLS-DA model, and statistical significance was evaluated using Welch’s one-way ANOVA followed by the Games–Howell post hoc test. The high-response origin was determined according to the highest group mean response among WZ, GX, FJ and SC. These variables were used to interpret origin-related GC-IMS fingerprint differences.

## Data Availability

The data presented in this study are available within the article. Additional data are available from the corresponding author upon reasonable request.

## Abbreviations

The following abbreviations are used in this manuscript:

HS-GC-IMS	Headspace gas chromatography–ion mobility spectrometry
GC-IMS	Gas chromatography–ion mobility spectrometry
VOC	Volatile organic compound
PCA	Principal component analysis
PLS-DA	Partial least squares discriminant analysis
VIP	Variable importance in projection
RI	Retention index
RT	Retention time
DT	Drift time
WZ	Wenzhou
GX	Guangxi
FJ	Fujian
SC	Sichuan
